# Matrine, a potential c-Myc inhibitor, suppresses ribosome biogenesis and nucleotide metabolism in myeloid leukemia

**DOI:** 10.3389/fphar.2022.1027441

**Published:** 2022-10-21

**Authors:** Wang-Jing Zhong, Lingdi Ma, Fanfan Yang, Jialin Cao, Junyu Tan, Bohong Li

**Affiliations:** ^1^ Laboratory Center, Huizhou Third People’s Hospital, Affiliated Hospital of Guangzhou Medical University, Huizhou, China; ^2^ Laboratory Medicine Department, Longhua Center for Chronic Disease Control, Shenzhen, China

**Keywords:** matrine, c-Myc, ribosome biogenesis, nucleotide metabolism, myeloid leukemia

## Abstract

Previous studies have shown that matrine, a natural compound extracted from the herb *Sophora flavescens*, has a good anti-leukemia effect, but its key target and mechanism remains unclear. Here, we found that only c-Myc could respond rapidly to matrine treatment in three myeloid leukemia cell lines, and matrine inhibited both transcription and translation of c-Myc. Ribosome biogenesis and nucleotide metabolism, the key downstream of c-Myc, were significantly suppressed after matrine treatment. Therefore, our results confirmed that matrine is a special c-Myc inhibitor which suppresses ribosome biogenesis and nucleotide metabolism by inhibiting c-Myc in myeloid leukemia. This study provides scientific basis for the development of matrine derivatives to c-Myc-driven cancers.

## Introduction

Abnormal activation of c-Myc is one of the most common features of human cancers. Overexpression of c-Myc is found in more than 50% of human cancers and is associated with poor prognosis (Dang, 2012; Wang et al., 2021). The occurrence and development of a variety of hematological cancers, such as leukemia (Calabretta and Skorski, 1997), lymphoma (Zhou et al., 2014) and multiple myeloma (Jovanović et al., 2018), are also associated with abnormal expression of c-Myc. As a transcription factor, c-Myc can regulate the expression of 15% of genes in the whole genome and regulate cell proliferation (Bouchard et al., 1998), apoptosis (Hoffman and Liebermann, 1998), differentiation (Henriksson and Lüscher, 1996), metabolism (Dejure and Eilers, 2017; Dong et al., 2020) and immune response (Casey et al., 2016; Casey et al., 2018). Although c-Myc is a potential anticancer target, it is difficult to be directly targeted by small molecule drugs because it is a natural disordered protein and lacks a suitable drug binding site. At present, c-Myc is mainly targeted indirectly. It is mainly achieved by inhibiting the transcription, translation, transcriptional activity and promoting ubiquitination and degradation of c-Myc, as well as inducing lethal synthesis (Chen et al., 2018). Among them, targeting bromine domain protein 4 (BRD4), a member of bromodomain and extraterminal (BET) fanmily, is the most promising method for inhibiting c-Myc. BRD4 inhibitors have been shown good anti-tumor effects in a variety of tumors, including acute myeloid leukemia and multiple myeloma, and a large number of BRD4 inhibitors are currently in clinical trials (Delmore et al., 2011; Zuber et al., 2011; Lu et al., 2020). Butler et al. recently found that the Lon protease from uropathogenic *Escherichia coli* (UPEC) directly binds c-Myc protein and degrades it (Butler et al., 2021), suggesting that c-Myc is not undruggable. The research of targeting c-Myc still faces many difficulties, such as drug resistance of small molecule inhibitors, and some inhibitors cannot be carried out in clinical trials due to large side effects. Therefore, it is a long-term and arduous task to find safer and more efficient c-Myc inhibitors.

Regulation of ribosome biogenesis is considered to be the core and oldest function of c-Myc. c-Myc regulates the production of rRNA through RNA polymerase I and promotes the transcription of ribosomal proteins and translation initiation factors mediated by RNA polymerase II. c-Myc also promotes RNA polymerase III-mediated tRNA biogenesis, which promotes ribosome biogenesis, which in turn affects mRNA translation and protein synthesis, and ultimately affects cell growth (van Riggelen et al., 2010; Destefanis et al., 2020). CX-5461, an RNA polymerase I inhibitor developed for ribosome biogenesis, has shown good stabilization in patients with diffuse large B-cell lymphoma and myeloma in phase I clinical trials (Nadine et al., 2017; Khot et al., 2019). In addition, c-Myc is an important regulator of nucleotide metabolism. It can directly regulate the expression of enzymes related to nucleotide synthesis, such as PRPS2, PPAT, PAICS, GRAT, UMPS and DHODH, which regulate the synthesis of purine and pyrimidine, thus playing an important role in cell metabolism and growth (Liu et al., 2008; Cunningham et al., 2014).

Matrine is an active alkaloid extracted from *Sophora flavescens*, which has anti-inflammatory, antiviral and anti-tumor activities (Sun et al., 2022). Our group has been committed to studying the anti-leukemia effect and underlying mechanism of matrine. Combined with previous studies by other researchers, matrine can inhibit the proliferation, induce apoptosis, autophagy and differentiation of leukemia cell lines, and regulate immune functions (Ma et al., 2015a; Zhang et al., 2015; Wu et al., 2017a; Wu et al., 2018), indicating that matrine has the potential to become a drug for the treatment of leukemia. At present, the molecular mechanism of matrine in leukemia mainly includes up-regulation of the expression of cell cycle suppressors such as P21 and P27, pro-apoptotic proteins, and cell surface differentiation antigens such as CD11b and CD15, down-regulation of oncogenic genes such as c-Myc, HK2, CyclinD1, MMP, HIF-1α, VEGF and anti-apoptotic proteins, modulation of ERK/MAPK, PI3K/Akt/mTOR, IL-6/JAK/STAT3 and other pathways, regulates mitochondrial function and phospholipase cPLA2 activity, as well as the expression of related inflammatory factors and adhesion molecules (Ma et al., 2015b; Wu et al., 2017a; Ma et al., 2017; Lin et al., 2019). In general, matrine can regulate the expression of multiple genes and regulate multiple signaling pathways, but its key targets and main mechanism are still unclear. Therefore, finding the most critical target of matrine is the key to reveal the important mechanism of matrine against leukemia.

Our group previously found that matrine can inhibit the expression of c-Myc in leukemia cell lines, and found that matrine regulates the expression of glycolysis-related enzyme HK2, which may be related to c-Myc (Ma et al., 2017; Lin et al., 2019). Here, we found that c-Myc was not only regulated by matrine, but also the only molecule that responded rapidly to matrine treatment. Matrine inhibited gene transcription, ribosome biogenesis and nucleotide metabolism by inhibiting both c-Myc transcription and translation. Our results suggest that matrine may be a novel c-Myc inhibitor and may have great potential for treating c-Myc-driven cancers.

## Materials and methods

### Primary leukemia cells and normal PBMCs

Peripheral blood mononuclear cells (PBMCs) were isolated from peripheral blood collected from a patient diagnosed with acute myeloid leukemia (AML M5) in Huizhou Central People’s Hospital and six healthy subjects. No treatment with radiation or chemotherapy had been conducted before peripheral blood collection. Ficoll reagent was used to isolate PBMCs in peripheral blood collected within 1 h. Informed consent was obtained from each subject, and the protocol was approved by the Institutional Research Ethics Committee of Huizhou Third People’s Hospital.

### Cell culture

Human Acute Myeloid Leukaemia cell lines HL-60 and U937 and Chronic Myeloid Leukemia cell lines K562 were obtained from the Cell Bank of Chinese Academy of Sciences (Shanghai, China). All of cell lines were culture in RPMI 1640 medium with 10% fetal bovine serum (164210, Procell, China) and 1% penicillin/streptomycin (SV30010, Hyclone, USA).

### Reagents and antibodies

Matrine (S2322, Selleck, USA) was dissolved in ddH_2_O with a final concentration of 40 mg/ml and stored at +4°C. ddH_2_O was used as vehicle control for matrine. Other reagents and antibodies used included: Cycloheximide (CHX, S7418, Selleck, USA), MG132 (S2619, Selleck, USA), Actinomycin D (ACTD, S8964, Selleck, USA), cell counting kit-8 (CCK-8, CK04, Dojindo, Japan), Anti-c-Myc (10828-1-AP, Proteintech group, USA) and Anti-β-actin (23660-1-AP, Proteintech group, USA). DMSO as control for CHX, MG132, and ACTD.

### Cell viability analysis

The cell viability was detected by the Cell Counting Kit-8 (CCK-8) assay. Briefly, U937, HL-60 and K562 cel lines were seeded into the 96-well plate and treated with matrine. 10μL of CCK-8 solution was added into the medium and cultured for 4 h at 37°C before detection. The absorbance of each well at 450 nm was detected by the microplate reader (iMark, Bio-Rad, America).

### RNA isolation and real-time quantitative PCR

Total RNA of Leukemia cells was extracted using Trizol reagent (R0016, Beyotime Biotechnology, China) and reversely transcribed into complementary DNA using ReverTra Ace® qPCR RT Master Mix with gDNA remover (FSQ-301, TOYOBO, Japan). cDNA was quantified by qPCR on a 7,500 real-time PCR System (Thermo Fisher Scientific, USA) using a SYBR Green qPCR kit (522076, Bimake, USA). Relative expression of every gene was calculated by 2^−ΔΔCt^ method and normalized to β-actin. Primers of all genes were designed using Primer 3 software and synthesized in Tsingke Biotechnology. The sequences of the primers are in Supplementary Table S11.

### Protein extraction and western blotting

Total proteins of Leukemia cells were lysed in RIPA buffer (P0013B, Beyotime Biotechnology, China) and total proteins were separated on 10% SDS-PAGE gels, electrophoretically transferred onto polyvinylidene difluoride membranes (IPVH00010, Milipore, USA), then Incubated with antibodies at 4°C overnight after blocking by 5% skimmed milk. After incubation with HRP-coupled secondary antibody, protein signal was detected by chemiluminescence reagent (ECL, Beyotime Biotechnology, China) using high sensitivity chemiluminescence imaging system (Gel Doc™ XR+, Bio-Rad, USA) and quantitatively analyzed by image J software.

### Construction of stable cell lines

Construction of c-Myc-overexpressing cell lines: Lentivirus Specifically adapted for suspension cell lines was purchased from Genechem (Shanghai, China), which contains a lentiviral vector made up of pRRLSIN-cPPT-SFFV-MCS-3Flag-E2A-EGFP-SV40-puromycin element. The CDS sequence of ENST00000377970.6 transcript of c-Myc was selected for vector construction and virus packaging. The cells were seeded in 12-well plates 1 day in advance, infected with a virus content of MOI equal to 30, and added with 30 µL HitransB-2 infection enhancement solution. The cells were cultured at 37°C for 12–16 h and then replaced with fresh medium. After 72 h of infection, the infection efficiency was observed with fluorescence microscope (DMI8, Leica, Germany).

Construction of monoclonal c-Myc-knockout cell lines: Stable monoclonal c-Myc-knockout cell lines was constructed by Haixing Biotechnology (Hunan, China). A lentiviral vector containing double sgRNA that specifically targets the ENST00000377970.6 transcript of c-Myc gene was constructed. The sgRNA expression vector was then electrically transferred into cells by Neon® transfection system (Thermo Fisher Scientific, USA). After purinomycin screening, the cells were seeded into 96-well plates for monoclonal growth. When the cells grew to a certain number, genomic DNA was extracted for PCR reaction to identify the knockout effect. The sequences of the gRNAs are in Supplementary Table S11.

### Global nascent proteins detection

Protein Synthesis Assay Kit (ab239725, Abcam, UK) was used for the detection of global nascent proteins of leukemia cells following the instructions of the manufacturer. Briefly, cells were collected after replacement of fresh medium with protein label for 2 h. After fixation and permeability, the reaction cocktail was added and incubated with the cells away from light for 30 min. Then the cells were washed with 1×PBS and detected by flow cytometry (FACSCanto, BD, USA).

### RNA sequencing

After extraction, total RNA of each sample was quantified and qualified to inspect RNA integrity by Agilent 2,100 Bioanalyzer (Agilent Technologies, USA). Next generation sequencing library preparations were constructed according to the manufacturer’s protocol. The poly(A) mRNA isolation was performed using Poly (A) mRNA Magnetic Isolation Module or rRNA removal Kit. Then libraries with different indices were multiplexed and loaded on an Illumina HiSeq instrument according to manufacturer’s instructions (Illumina, USA). Sequencing was carried out using a 2 × 150bp paired-end (PE) configuration. The sequences were processed and analyzed by GENEWIZ. RNA-seq reads was mapped to the human genome reference assembly (hg38) using Hisat2 (v2.0.1). HTSeq (v0.6.1) was used to estimated gene and isoform expression levels. Significant differential expressed genes were identified as those with a adjust *p* value above the threshold (Padj<0.05), foldchange>1.5 or foldchange>1.2 and FPKM>1 using DESeq2 software.

### LC-MS/MS untargeted metabolomics

Metabolomics was used to evaluate the effect of matrine on nucleotide metabolism performed by Luming Biology (Shanghai, China). Briefly, at least 10 million cells per sample are collected in biological triplicate and extracted with methanol-acetonitrile-water (V:V:V = 2:2:1) according to the manufacturer’s instructions, The metabolites were separated using liquid chromatography-mass spectrometry (LC-MS) in combination with HILIC chromatographic system, a chromatographic technique with the opposite elution sequence from RPLC to better isolate and identify strongly polar and hydrophilic compounds. A Nexera UPLC system fitted with Q-Exactive plus quadrupole-Orbitrap mass spectrometer equipped with heated electrospray ionization (HESI) source (Thermo Fisher Scientific, USA) was used to analyze the metabolic profiling in both HESI positive and HESI negative ion modes. The acquired LC-MS raw data were processed by software Compound Discoverer 3.0 for baseline filtering, peak identification, integral, and peak alignment. Compound identification were based on precise mass-to-charge ratio (M/z), secondary fragments, and isotopic distribution using standard product self-built databases to do qualitative analysis.

### Cell cycle assay

The cells of control group and treatment group were collected by centrifugation, and washed twice with pre-cooled 1×PBS, then fixed at 4°C overnight with pre-cooled 70% ethanol. The cells were collected by centrifugation and washed once with 1 ml 1×PBS. 500uL 1×PBS containing 50ug/mL PROpyl bromide (PI), 100 ug/mL RNase A and 0.2% Triton X-100 was added and incubated at 4°C for 30 min under dark conditions. Flow cytometry (calibur, BD, USA) was used for detection and results were analyzed by the software ModFit.

### Time-course analysis of c-Myc degradation or accumulation

Cells undergoing matrine treatment were subjected to cycloheximide (CHX, 50 mg/ml) or Actinomycin D (ACTD, 2.5 µM) or MG132 (10 µM) and harvested at specific time-points. Total RNA or total proteins was then extracted and subjected to qPCR or immunoblot. β-actin was used as an internal control. Protein band densities were quantified by image J software.

### Bioinformatics

For data acquisition and processing, SLAM-Seq data of c-Myc knockdown by Auxin-Inducible Degron (AID) System came from GEO Datasets (GSE111457), Significant differential expressed genes (DEGs) were identified as those with a adjective *p* value (Padj) above the threshold (Padj<0.05), foldchange>1.5; Seven Chip-Seq datasets of MYC of K562 cell lines was obtained from Cistrome Database (GSM748551, GSM748553, GSM748552, GSM822310, GSM935516, GSM935410 and GSM487427). The intersection of MYC potential targets (score ≥1) from seven Chip-Seq datasets was performed.

Gene Set Enrichment Analysis (GSEA) were performed using GSEA (v.4.2.3) within MSigDB 7.5.1 (http://www.gsea-msigdb.org/gsea/index.jsp). Kyoto Encyclopedia of Genes and Genomes (KEGG) enrichment analysis were performed using the OECloud tools (v.1.2.12) (https://cloud.oebiotech.cn).

### Statistical analysis

The intersection of two or more groups was performed by INTERSECT function using R 4.0.3. Fower plot and violin diagram were drawn by R packages Plotrix (v.3.8.2) and Vioplot (v.0.3.7). Venn diagram was drawn using a software tool (http://bioinformatics.psb.ugent.be/webtools/Venn/). All other statistical analyses were performed using GraphPad Prism version 7.0.4 (GraphPad Software Inc., USA). Data in this study are shown as mean ± SEM from at least three independent replicates. Unpaired t-test or two-way ANOVA was carried out to compare the significant differences between the two groups. *p* values < 0.05 was considered significant difference, and all statistical tests in this study were two-sided tests.

## Results

### Matrine can rapidly regulate the expression of c-Myc

**FIGURE 1:**
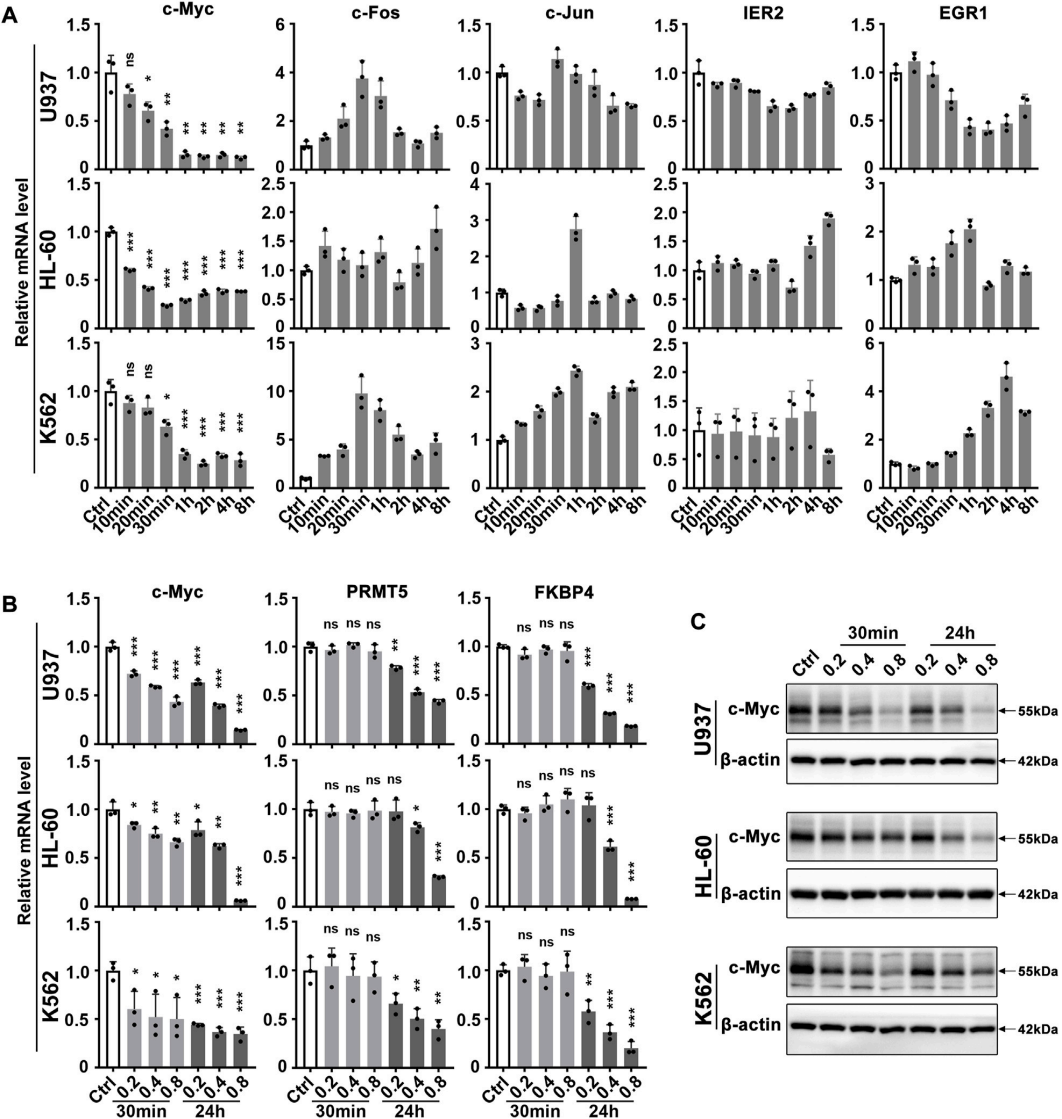
c-Myc responds rapidly to matrine treatment. **(A)** Effects of matrine on mRNA levels of immediate early genes in myeloid leukemia cell lines. U937, HL-60 and K562 cell lines were treated with 0.8 mg/ml matrine for different time and then subjected to qPCR. **(B,C)** Effects of matrine on expression of c-Myc and its targets. U937, HL-60 and K562 cell lines were treated with 0.2, 0.4 and 0.8 mg/ml matrine for 30 min and 24h, respectively, and then subjected to qPCR **(B)** or western blotting **(C)**. β-actin was used as an internal control. Data are shown as mean ± SEM of three independent replicates and unpaired t-test was carried out to detect significance **(A,B)**. ns, *p < 0.05, **p < 0.01, ***p < 0.001 vs. Ctrl. Abbreviations: Ctrl, control; ns, no significance.

Our group and others have previously reported that matrine has a good anti-leukemia effect. Here we further explore its key targets and mechanisms using several myeloid leukemia cell lines. To investigate which genes respond rapidly to matrine treatment, we examined the expression of immediate early genes that are closely related to cell growth. To our surprise, it was c-Myc that responded rapidly to matrine treatment, and matrine reduced the mRNA levels of c-Myc without significantly affecting other early genes in U937, HL-60 (acute myeloid leukemia cell lines) and K562 cell lines (chronic myeloid leukemia cell lines) (Figure 1A). QPCR and western blotting results further showed that c-Myc could respond to matrine treatment more rapidly than its targets PRMT5 (Koh et al., 2015) and FKBP4 (Zeller et al., 2003) (Figures 1B,C). Morecver, we found that matrine treatment for 30min or 24 h did not significantly affect the mRNA levels of MYCN and MYCL, two other members of the MYC family, except that matrine reduced the mRNA levels of MYCL in U937 cells (Supplementary Figure S1). These results suggest that matrine can rapidly regulate the expression of c-Myc.

### Matrine may inhibit the proliferation of myeloid leukemia cell lines by downregulating the expression of c-Myc

**FIGURE 2:**
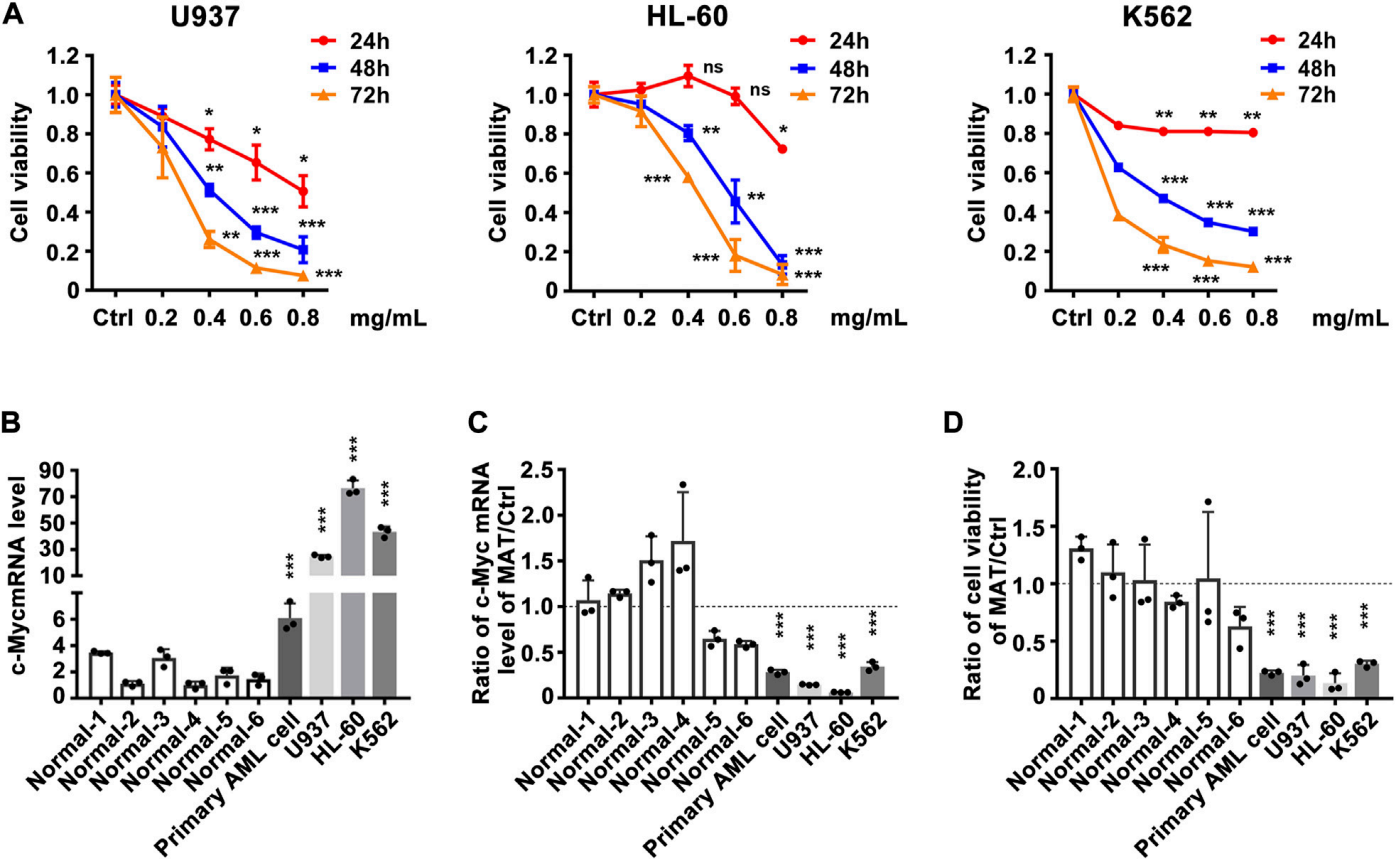
The inhibitory effect of matrine on the proliferation of myeloid leukemia cell lines is related to the expression inhibition of c-Myc. **(A)** Effects of matrine on proliferation of myeloid leukemia cell lines. U937, HL-60 and K562 cell lines were treated with different concentrations of matrine for 24h, 48 h and 72 h, respectively, and then subjected to CCK-8 assay. **(B)** Comparison of c-Myc expression levels between normal peripheral blood mononuclear cells (PBMCs), primary leukemia cells and myeloid leukemia cell lines. **(C)** Comparison of the inhibitory effect of matrine on c-Myc expression in PBMCs, primary leukemia cells and myeloid leukemia cell lines. **(D)** Comparison of the inhibitory effect of matrine on proliferation in PBMCs, primary leukemia cells and myeloid leukemia cell lines. c-Myc mRNA levels were detected by qPCR **(B,C)**. Cell viability was detected by CCK-8 assay **(D)**. β-actin was used as an internal control. Data are shown as mean ± SEM of three independently triplicates and unpaired t-test was carried out to detect significance **(B–D)**. ns, *p < 0.05, **p < 0.01, ***p < 0.001 vs. Ctrl **(A)** or the mean of six normal subjects **(B–D)**. Ctrl, control; ns, significance; Normal 1-6, normal subjects, AML, Acute Myeloid Leukemia.

Since matrine can regulate c-Myc expression, does matrine exerts its anti-leukemia effect through c-Myc? We determined that matrine could inhibit the proliferation of U937, HL-60 and K562 cell lines in a time- and concentration-dependent manner (Figure 2A). c-Myc expression was significantly increased in myeloid leukemia cell lines and primary acute myeloid leukemia (AML) cells compared with PBMCs of normal subjects (Figure 2B), and matrine inhibited the expression of c-Myc and proliferation of myeloid leukemia and primary AML cells more significantly (Figures 2C,D). These results suggest that matrine may inhibit the proliferation of myeloid leukemia cells by inhibiting the expression of c-Myc.

### c-Myc may be the most critical target of matrine

**FIGURE 3:**
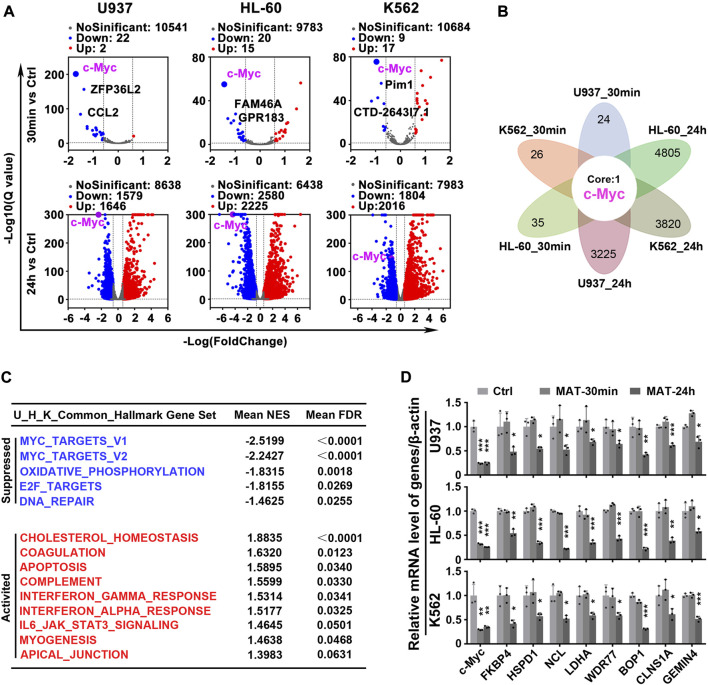
Matrine mainly targets c-Myc to regulate gene transcription. **(A)** Volcano plot of genes from RNA-Seq of Myeloid leukemia cell lines. U937, HL-60 and K562 cell lines were treated with 0.8 mg/ml matrine for 30 min and 24 h, respectively, and then subjected to mRNA sequencing. Down and Up, differentially expressed gene, |Foldchange| ≥ 1.5 and padj <0.05 and FPKM>1. **(B)** The intersection of differentially expressed genes from RNA-seq of 3 cell lines treated with matrine. **(C)** Hallmark GSEA analysis was used for the genes from RNA-Seq of U937,HL-60 and K562 cell lines treated with matrine, and then the intersection of the 3 cell lines was obtained. The NES and FDR values of the pathways were ranked by means. **(D)** QPCR analysis of representative c-Myc targets in 3 cell lines treated with 0.8 mg/ml matrine for 30min and 24 h. β-actin was used as an internal control. Data are shown as mean ± SEM of three independently triplicates. *p < 0.05, **p < 0.01, ***p < 0.001 vs. Ctrl, unpaired t-test. MAT, matrine.

To test the hypothesis that matrine will specifically abrogate c-Myc-dependent transcription, we utilized global transcriptional profiling. We first characterized the transcriptome consequences of three myeloid leukemia cell lines treated with matrine for 30 min and 24 h (Figure 3A). Surprisingly, after matrine treatment for 30 min and 24 h, only c-Myc was significantly changed in all 3 cell lines simultaneously (Figure 3B). Gene set enrichment analysis (GSEA) (Subramanian et al., 2005) found that c-Myc target gene sets was most significantly suppressed (Figure 3C, Supplementary Figures S2A,B, Supplementary Tables S1, S2). Comparison with the database of c-Myc target genes by qPCR confirmed that matrine treatment reduced expression of many such genes (Figure 3D). These results suggest that c-Myc may be the key target of matrine and matrine mainly inhibited c-Myc-dependent transcription.

### Matrine suppresses ribosome biogenesis and nucleotide metabolism

**FIGURE 4:**
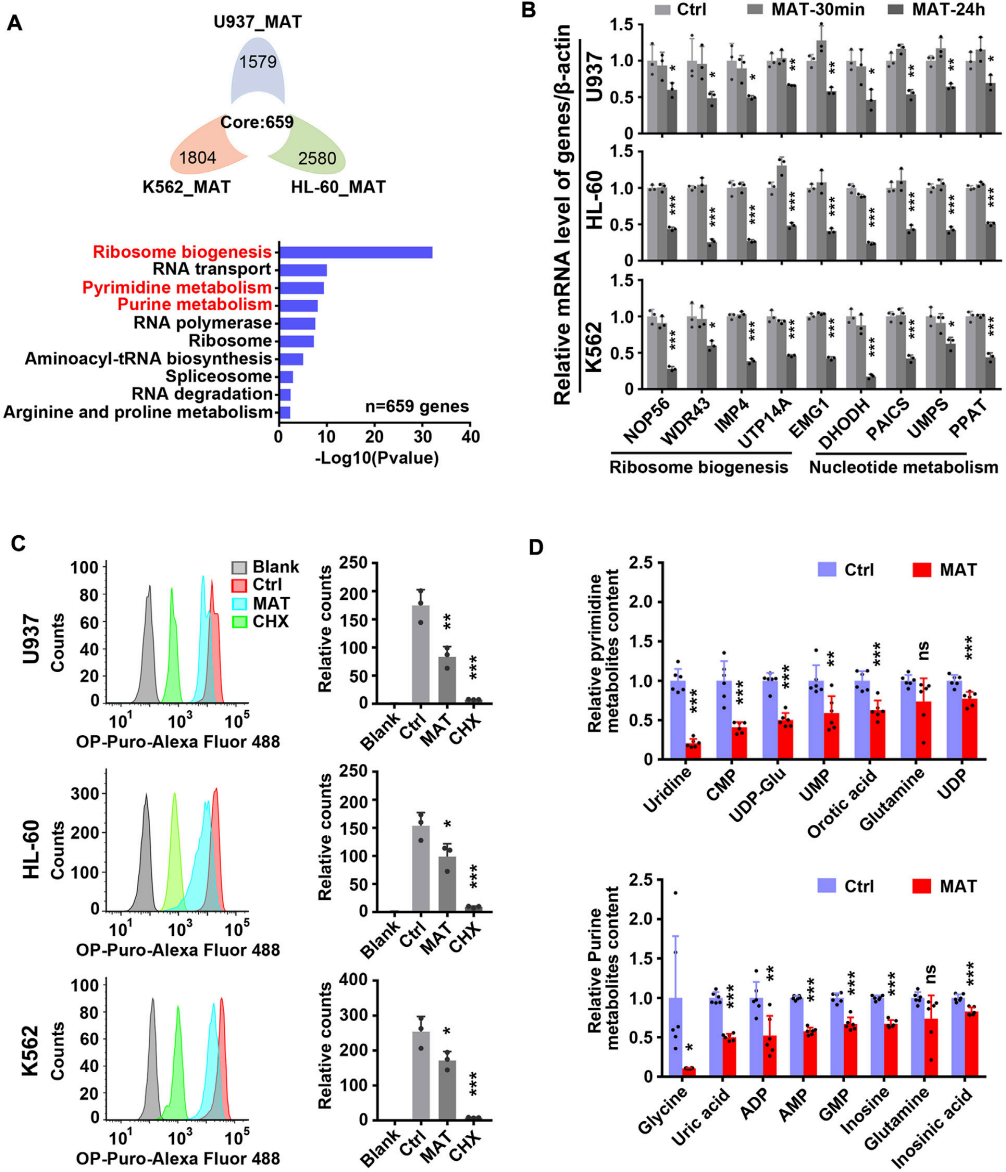
Matrine suppresses ribosome biogenesis and nucleotide metabolism. **(A)** The intersection and KEGG enrichment analysis of genes down-regulated (|Foldchange| ≥ 1.5, padj <0.05) in RNA-Seq of 3 cell lines treated with 0.8 mg/ml matrine for 24 h. Flower plot (above) and KEGG enrichment analysis (below) are shown.**(B)** QPCR analysis of representative ribosome biogenesis and nucleotide metabolis-related genes in 3 cell lines treated with 0.8 mg/ml matrine for 30 min and 24 h. **(C)** Measurement of global nascent protein synthesis by means of O-Propargyl-puromycin (OP-Puro) incorporation and flow cytometry in 3 cell lines treated with 0.8 mg/ml matrine for 24 h or 50ug/mL CHX for 2 h. Representative image (left) and quantified level of global protein synthesis (right) are shown. **(D)** LC-MS/MS metabolomic analysis (HILIC) for pyrimidine and purine metabolism-related substances of U937 cell line treated with 0.8 mg/ml matrine for 24 h. Data are shown as mean ± SEM of three independently triplicates **(B,C)** or six independently triplicates **(D)**. Significance determined for each cell line treated with matrine or CHX vs. Ctrl. *p < 0.05, **p < 0.01, ***p < 0.001, unpaired t-test. CMP, cytidine 5′-monophosphate; UDP-Glu, uridine 5′-diphosphoglucose; UMP, uridine 5′-monophosphate; UDP, uridine 5′-diphosphate; ADP, adenosine 5′-diphosphate; AMP, adenosine 5′-monophosphate; GMP, guanosine 5′-monophosphate.

Further, we analyzed the effect of matrine on global gene expression. The transcriptome results showed that 659 genes were down-regulated and 382 genes were up-regulated simultaneously in three leukemia cell lines treated with matrine for 24 h (Figure 4A above and Supplementary Figure S4 left). Kyoto Encyclopedia of Genes and Genomes (KEGG) enrichment analysis of down-regulated genes showed that ribosome biogenesis and nucleotide metabolism were significantly enriched, suggesting that matrine mainly inhibits the two pathways (Figure 4A below, Supplementary Figures S3A,B, Supplementary Tables S3–S5). QPCR results conformed that matrine inhibited the expression of genes related to ribosome biogenesis and nucleotide metabolism (Figure 4B). Correspondingly, matrine treatment inhibited intracellular global protein synthesis, although not as significantly as positive control cycloheximide (CHX) (Figure 4C). We also performed metabolomics and found that matrine did significantly reduce the content of substances related to pyrimidine and purine metabolism (Figure 4D). These results suggest that matrine may play an anti-leukemia role by targeting ribosome biogenesis and nucleotide metabolism pathways.

### c-Myc may mediate the inhibitory effect of matrine on ribosome biogenesis and nucleotide metabolism

**FIGURE 5:**
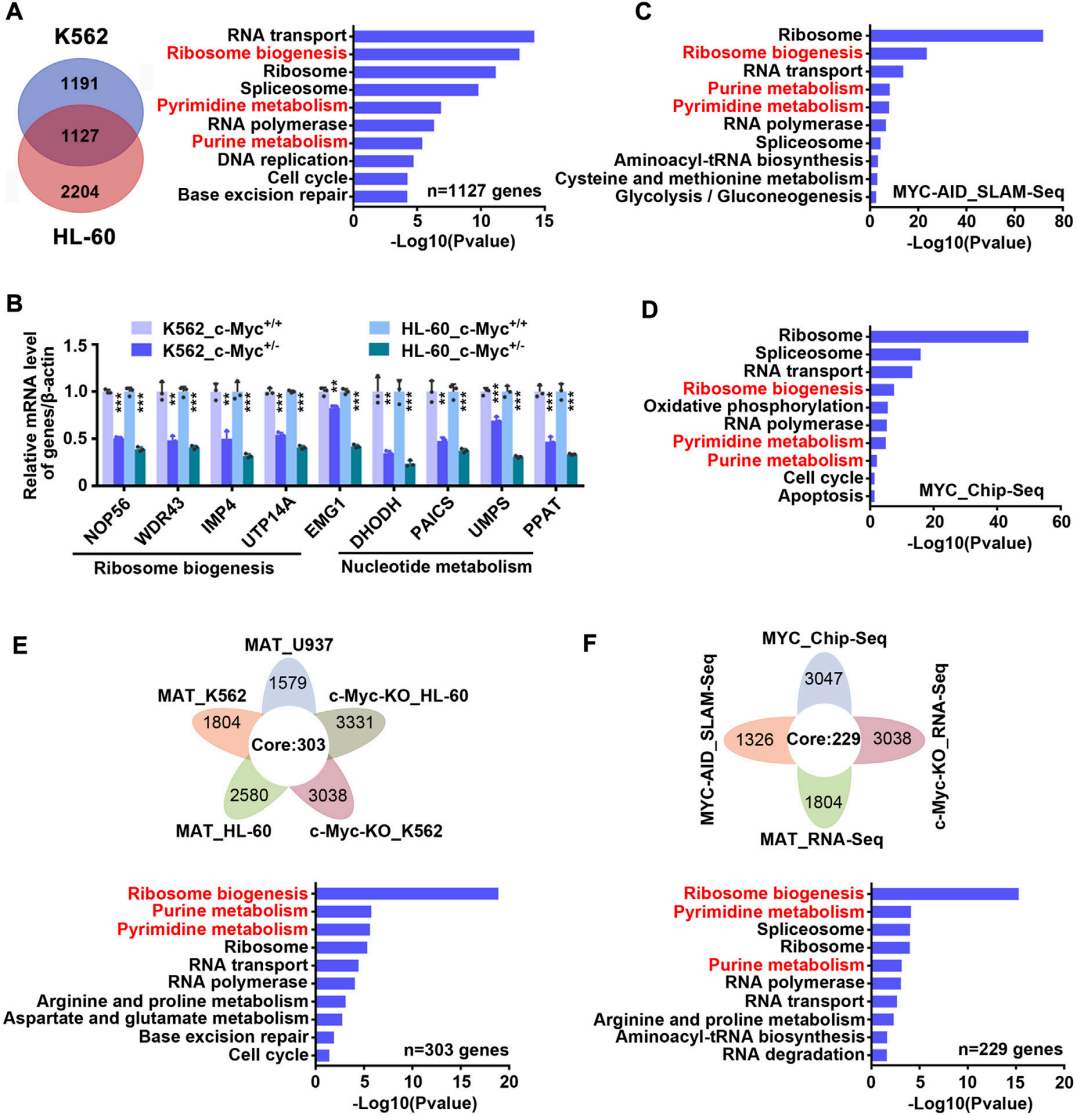
Matrine may suppress both ribosome biogenesis and nucleotide metabolism through c-Myc. **(A)** The intersection and KEGG enrichment analysis of genes down-regulated (|Foldchange| ≥ 1.2, padj <0.05) in RNA-Seq of K562 and HL-60 cell lines upon c-Myc heterozygous knockout. The intersection (left) and KEGG enrichment analysis (right) are shown. **(B)** KEGG enrichment analysis for down-regulated genes (|Foldchange| ≥ 1.2, padj <0.05) upon MYC depletion by AID system (GSE111457). **(C)** QPCR analysis of representative ribosome biogenesis and nucleotide metabolis-related genes in K562 and HL-60 cell lines upon c-Myc knockout. β-actin was used as an internal control. Data are shown as mean ± SEM of three independently triplicates. *p < 0.05, **p < 0.01, ***p < 0.001, unpaired t-test. **(D)** The intersection and KEGG enrichment analysis of MYC potential targets (score ≥1) from seven Chip-Seq datasets (GSM748551, GSM748553, GSM748552, GSM822310, GSM935516, GSM935410, GSM487427, from Cistrome database) in K562 cell line. **(E)** The intersection and KEGG enrichment analysis of down-regulated genes from RNA-Seq of U937,HL-60 and K562 cell lines treated with matrine and down-regulated genes from RNA-Seq of HL-60 and K562 cell lines upon c-Myc knockout. **(F)** The intersection and KEGG enrichment analysis of down-regulated genes from MAT_RNA-Seq, c-Myc-KO_RNA-Seq, MYC-AID_SLAM-Seq and MYC potential targets from MYC_Chip-seq in K562 cell line. Flower plot (above) and KEGG enrichment analysis (below) are shown **(E,F)**.

c-Myc is considered to be an important regulator of ribosome biogenesis and nucleotide metabolism. In order to verify whether c-Myc can regulate these two pathways in our model, we constructed monoclonal cell lines of c-Myc-knockout heterozygote using CRISPR-Cas9 technology in K562 and HL-60 cell lines (Supplementary Figures S5A–C), and then carried out transcriptome sequencing. It was found that 1,127 genes were down-regulated and 860 genes were up-regulated simultaneously in K562 and HL-60 cell lines after knocking out c-Myc (Figure 5A left and S6 left, Supplementary Table S6). KEGG enrichment analysis of down-regulated genes showed that ribosome biogenesis and nucleotide metabolism were significantly inhibited (Figure 5A right, Supplementary Table S6), while up-regulated genes were mainly enriched in lysosome and other pathways (Supplementary Figure S6 right, Supplementary Table S6). QPCR results also verified that c-Myc targets were down-regulated after knocking out c-Myc (Figure 5B). Analysis of the SLAM-seq consequence of c-Myc knockdown by Auxin-Inducible Degron (AID) system (Muhar et al., 2018) found that ribosome biogenesis and nucleotide metabolism were also significantly inhibited (Figure 5C and Supplementary Table S7), which coincided with the effect of matrine. In addition, we also analyzed seven chip-seq datasets of MYC in K562 cell line from cistrome database, and got 3,047 genes with score >1 as potential targets of MYC, and subsequently found that these MYC targets could still be significantly enriched in ribosome biogenesis and nucleotide metabolism pathways (Figure 5D and Supplementary Table S8). These data suggest that c-Myc regulates ribosome biogenesis and nucleotide metabolism, and that both pathways are major downstream effects of c-Myc.

Based on the above results, we speculated that matrine regulates ribosome biogenesis and nucleotide metabolism through c-Myc. To test this, we integrated the transcriptome consequences of matrine treatment and c-Myc knockout, and found that 303 genes were down-regulated after matrine treatment and c-Myc knockout (Figure 5E above). KEGG enrichment analysis of these genes showed that ribosome biogenesis and nucleotide metabolism pathways were significantly enriched (Figure 5E below and Supplementary Table S9). Furthermore, we compared the two transcriptome data of K562 cells performed by our group with SLAM-Seq results of c-Myc knockdown and MYC Chip-Seq data performed by other groups, and obtained 258 intersected genes (Figure 5F above). KEGG enrichment analysis of these genes revealed that ribosome biogenesis and nucleotide metabolism were still significantly enriched (Figure 5F below and Supplementary Table S10). Therefore, these results suggest that matrine regulates ribosome biogenesis and nucleotide metabolism through c-Myc.

### Matrine inhibits both transcription and translation of c-Myc

**FIGURE 6:**
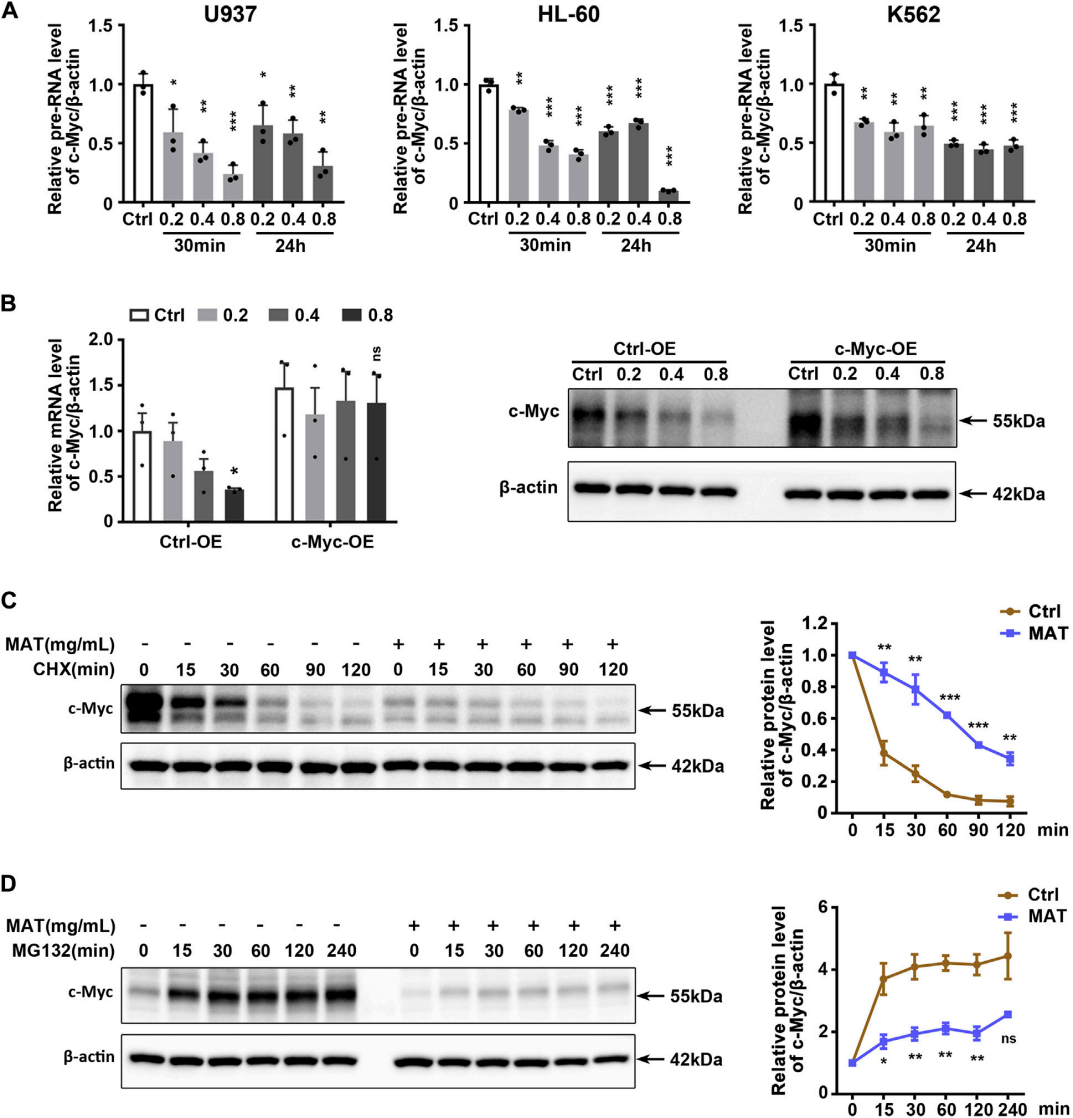
Matrine inhibits both transcription and translation of c-Myc. **(A)** QPCR analysis of pre-RNA level of c-Myc in 3 cell lines treated with 0.2,0.4 and 0.8 mg/ml matrine for 30 min and 24 h, respectively. **(B)** QPCR and western blotting analysis of c-Myc mRNA and protein level in c-Myc-overexpressing U937 cell line and control group treated with 0.2,0.4 and 0.8 mg/ml matrine for 24 h. **(C,D)** Time-course analysis of c-Myc protein level in U937 cell line. Cells were treated with 0.8 mg/ml matrine for 22 h **(C)** or 20 h **(D)**, then incubated with 50 μg/ml CHX **(C)** or 10 µM MG132 **(D)** before immunoblotting. Representative image (left) and quantified level of c-Myc (right) are shown. Data are shown as mean ± SEM of three independently triplicates. *p < 0.05, **p < 0.01, ***p < 0.001, unpaired t-test **(A,B)** or two-way ANOVA **(C,D)**.

Since matrine reduced c-Myc mRNA levels, we hypothesized that matrine might inhibit the transcription of c-Myc. QPCR results showed that matrine significantly reduced c-Myc precursor RNA (pre-RNA) levels (Figure 6A), which supported our hypothesis. However, we later found that overexpression of c-Myc could counteract the lowering effect of matrine on c-Myc mRNA levels, but not abolish the lowering effect of matrine on c-Myc protein levels (Figure 6B). Actually, overexpression of c-Myc did not antagonize the inhibitory effects of matrine on cell cycle and proliferation (Supplementary Figures S7A,B). Therefore, matrine may also inhibit the expression of c-Myc in ways other than transcriptional regulation. We performed mRNA stabilization experiments and found that matrine did not affect the mRNA stability of c-Myc (Supplementary Figure S8). Then, we used the translation inhibitor CHX to explore whether matrine promoted the protein degradation of c-Myc, and found that matrine did not promote the protein degradation of c-Myc, and even had an inhibiting trend (Figure 6C), while the experiment of proteasome inhibitor MG132 showed that matrine might inhibit the mRNA translation of c-Myc (Figure 6D). These results suggest that matrine inhibits both transcription and translation of c-Myc.

## Discussion

c-Myc is a exciting therapeutic target because it regulates not only the intrinsic growth of tumor cells, but also the host immune response. However, the current strategy of indirectly targeting c-Myc has not achieved sufficient success, so scientists still need to focus on finding more effective drugs or means to target c-Myc. Our study demonstrated that the key target of matrine monomer is c-Myc. Matrine could simultaneously inhibit c-Myc transcription and translation, and inhibit the downstream ribosome biogenesis and nucleotide metabolism, thereby killing myeloid leukemia cell lines, suggesting that matrine can be used as an inhibitor of c-Myc to treat c-Myc-driven leukemia.

Of concern, an important role of matrine has been found to regulate autophagy, although whether matrine promotes or inhibits autophagy is controversial (Wang et al., 2013; Wu et al., 2017a). Here, we also found that the genes up-regulated by matrine were significantly enriched in the lysosomal pathway (Supplementary Figure S4 right), indicating that the effect of matrine was related to the lysosome, which is also our future exploration direction. However, we were surprised to find that c-Myc is a key target of matrine, which inhibits c-Myc expression most rapidly and significantly. Although previous studies have shown that matrine can inhibit c-Myc expression (Ma et al., 2017; Lin et al., 2019; Gu et al., 2020; Gu et al., 2021), these studies were insufficient and did not highlight the key role of c-Myc in the effects of matrine. In addition, previous studies have found that matrine can inhibit the progression of leukemia through Akt/mTOR and other signaling pathways, but here we have found a more critical target and mechanism of matrine. c-Myc may also be a key target of matrine in other tumors, and we will further explore it in the future.

*In vitro* studies, we found that matrine can specifically target c-Myc in myeloid leukemia cells, and matrine exerts its anti-leukemic effect by inhibiting c-Myc, but these conclusions have not yet been tested *in vivo*, which is also a pity of this study. In addition, the effective concentration of matrine against leukemia *in vitro* reached millimolar level, so matrine may cause some side effects *in vivo* due to the excessive dosage. Our next study will focus on the development of more efficient matrine derivatives and elucidation of their anti-leukemia effect and mechanism *in vivo*. Matrine is an ideal lead compound with wide pharmacological activity, good safety and abundant source (You et al., 2020; Sun et al., 2022). Therefore, the researchers have used their wisdom to develop several matrine derivatives, such as MASM (M19) (Xu et al., 2014), WM130 (Qian et al., 2015), WM622 (Sun et al., 2018) and YF-18 (Wu et al., 2017b), which have shown stronger antitumor effects in tumors other than leukemia. If the key target of matrine can be clearly identified and its structure can be purposefully modified, it is of great significance to develop new matrine-derived antitumor drugs with high efficiency, specificity and low toxicity.

Many drugs reported can target c-Myc at multiple stages including transcription and post-translational levels (Chen et al., 2018; Wang et al., 2021). Few drugs can simultaneously target c-Myc transcription and translation, but matrine does it. We think that matrine inhibits c-Myc transcription and translation through two different mechanisms, which will be the subject of our next study. G-quadruplexes and BRD4, are considered to be key regulators of c-Myc transcription (Delmore et al., 2011; Zuber et al., 2011; Hu et al., 2019), and we will explore whether the regulation of c-Myc by matrine is related to them. Matrine may also regulate mTORC1 and EIF4A-mediated signaling pathways, which are key regulators of c-Myc translation (Wullschleger et al., 2006; Wiegering et al., 2015). In addition, there may be other mechanisms involved in matrine’s regulation of c-Myc transcription and translation, which need to be further identified. Elucidating how matrine regulates c-Myc can better promote clinical research and the development of related derivatives.

Many genes related to ribosome biogenesis are mutated and dysregulated in a variety of tumors, resulting in abnormal ribosome biogenesis (Domostegui et al., 2021; Figueiredo and McCarthy, 2021). It is therefore not surprising that drugs targeting ribosome biogenesis have proven to be powerful tools against multiple cancer types, particularly with the recent development of RNA polymerase I inhibitors (Bywater et al., 2012; Khot et al., 2019). Many drugs routinely prescribed in chemotherapy, such as doxorubicin, cisplatin and methotrexate, act at different steps of ribosome biogenesis in addition to ribosome maturation and assembly (Burger et al., 2010). Our study showed that ribosome biogenesis is a key downstream of c-Myc, and matrine killed myeloid leukemia cell lines by inhibiting c-Myc-mediated ribosome biogenesis, suggesting that matrine is also a ribosome biogenesis inhibitor.

Nucleotides, composed of purines and pyrimidines, are the main building blocks of genetic materials. They are essential substances for the biosynthesis of RNA and DNA, cell metabolism and growth. Nucleotide metabolism, which includes the biosynthesis and degradation of nucleotides, is often altered during tumorigenesis and progression due to dysregulated expression of metabolism-related enzymes. Cancer cells must synthesize and utilize large amounts of nucleotides and energy for RNA and DNA, and up-regulated *de novo* nucleotide metabolism enables rapid cell proliferation, suggesting nucleotide metabolism is a potential target for cancer treatment (Cunningham et al., 2014; Aird and Zhang, 2015; Lane and Fan, 2015). Recently, targeting nucleotide metabolism has been shown to be a novel approach to enhance cancer immunotherapy (Wu et al., 2022). Here, We found that matrine could inhibit the expression of genes related to nucleotide metabolism, and thus inhibiting the content of nucleotide metabolism-related substances, suggesting that nucleotide metabolism plays an important role in matrine’s anti-myeloid leukemia effect and matrine has the potential to be used in immunotherapy for the treatment of cancers with disturbed nucleotide metabolism.

In conclusion, matrine can inhibit both transcription and translation of c-Myc, suggesting that matrine is a novel c-Myc inhibitor. Matrine mainly targets ribosome biogenesis and nucleotide metabolism downstream of c-Myc, suggesting that these two pathways are key to the anti-myeloid leukemia effect of matrine. Next, we will focus on the specific mechanism by which matrine inhibits both transcription and translation of c-Myc.

## Data Availability

The datasets presented in this study can be found in online repositories. The names of the repository/repositories and accession number(s) can be found below: NCBI Gene Expression Omnibus (GEO), GSE201309.

## Glossary

c-Myc: v-myc avian myelocytomatosis viral oncogene homolog
c-Fos: FBJ murine osteosarcoma viral oncogene homolog
c-Jun: jun proto-oncogene
Fos: FBJ murine osteosarcoma viral oncogene homolog B
JunB: jun B proto-oncogene
PRMT5: protein arginine methyltransferase 5
FKBP4: FK506 binding protein 4
HSPD1: heat shock 60 kDa protein 1
NCL: nucleolin
LDHA: lactate dehydrogenase A
WDR77: WD repeat domain 77
BOP1: block of proliferation 1
CLNS1A: chloride channel, nucleotide-sensitive, 1A
GEMIN4: gem (nuclear organelle) associated protein 4
NOP56: Nucleolar protein 56
WDR43: WD repeat domain 43
IMP4: IMP U3 small nucleolar ribonucleoprotein 4
UTP14A: UTP14A small subunit processome component
EMG1: EMG1 N1-specific pseudouridine methyltransferase
DHODH: dihydroorotate dehydrogenase (quinone)
PAICS: phosphoribosylaminoimidazole carboxylase and phosphoribosylaminoimidazole succinocarboxamide synthase
UMPS: uridine monophosphate synthetase
PPAT: phosphoribosyl pyrophosphate amidotransferase
β-actin: actin beta
MAT: matrine
Ctrl: control
CHX: cycloheximide
MG132: proteasome inhibitor
ACTD: actinomycin d
KO: knock out
OE: overexpression
ns: not significant
CMP: cytidine 5′-monophosphate
UDP-Glu: uridine 5′-diphosphoglucose
UMP: uridine 5′-monophosphate
UDP: uridine 5′-diphosphate
ADP: adenosine 5′-diphosphate
AMP: adenosine 5′-monophosphate
GMP: guanosine 5′-monophosphate
GSEA: gene set enrichment analysis
KEGG: kyoto encyclopedia of genes and genomes
SLAM-Seq: thiol (SH)–linked alkylation for the metabolic sequencing of RNA
RNA-Seq: RNA sequencing
Chip-Seq: Chromatin Immunoprecipitation sequencing.
