# Supplementary figures and images for: Matrine, a potential c-Myc inhibitor, suppresses ribosome biogenesis and nucleotide metabolism in myeloid leukemia
Source: Front Pharmacol. 2022 Oct 21;13:1027441. doi: 10.3389/fphar.2022.1027441 (PMC9634663; doi:10.3389/fphar.2022.1027441)

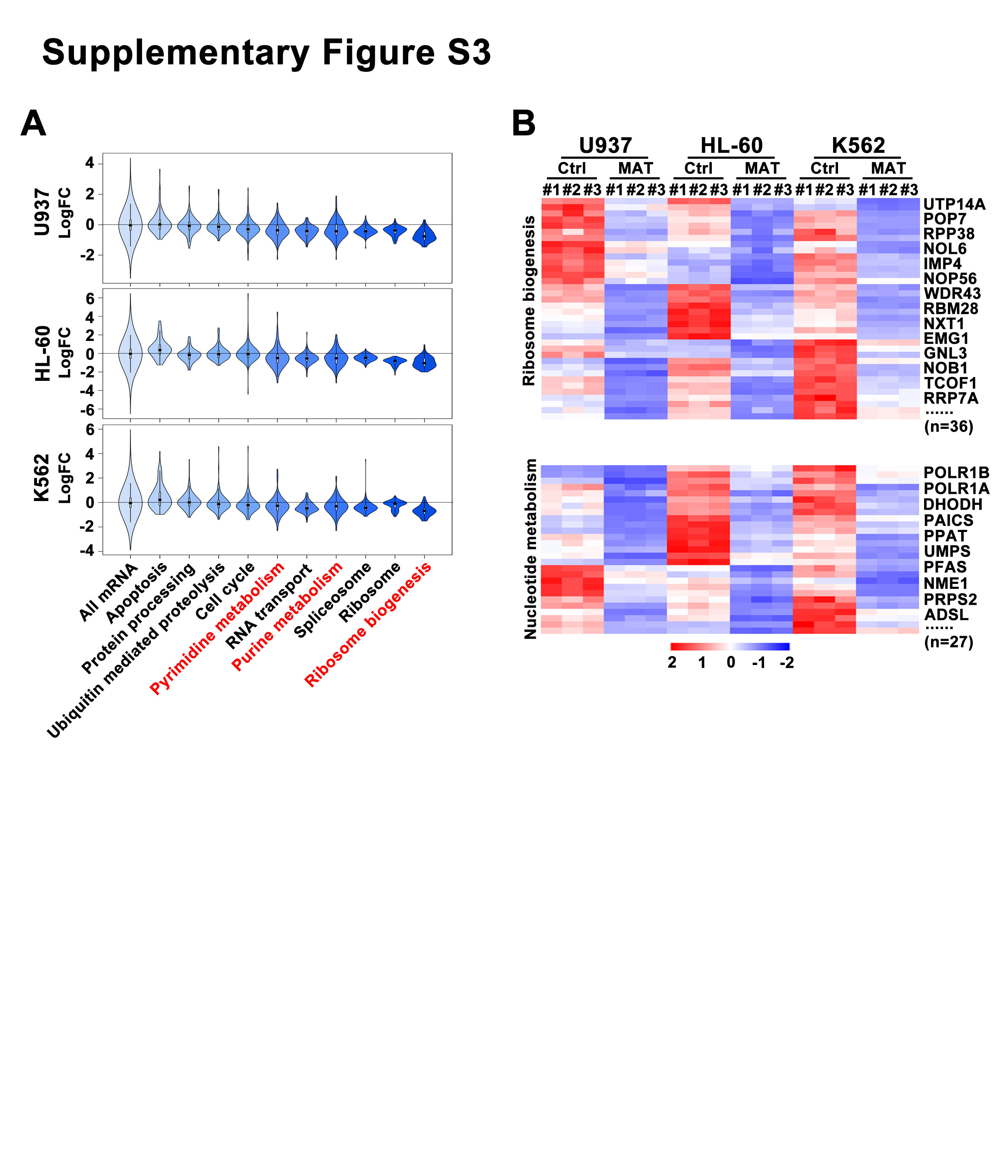

Supplement: Supplementary file 1 [file Image3.JPEG]

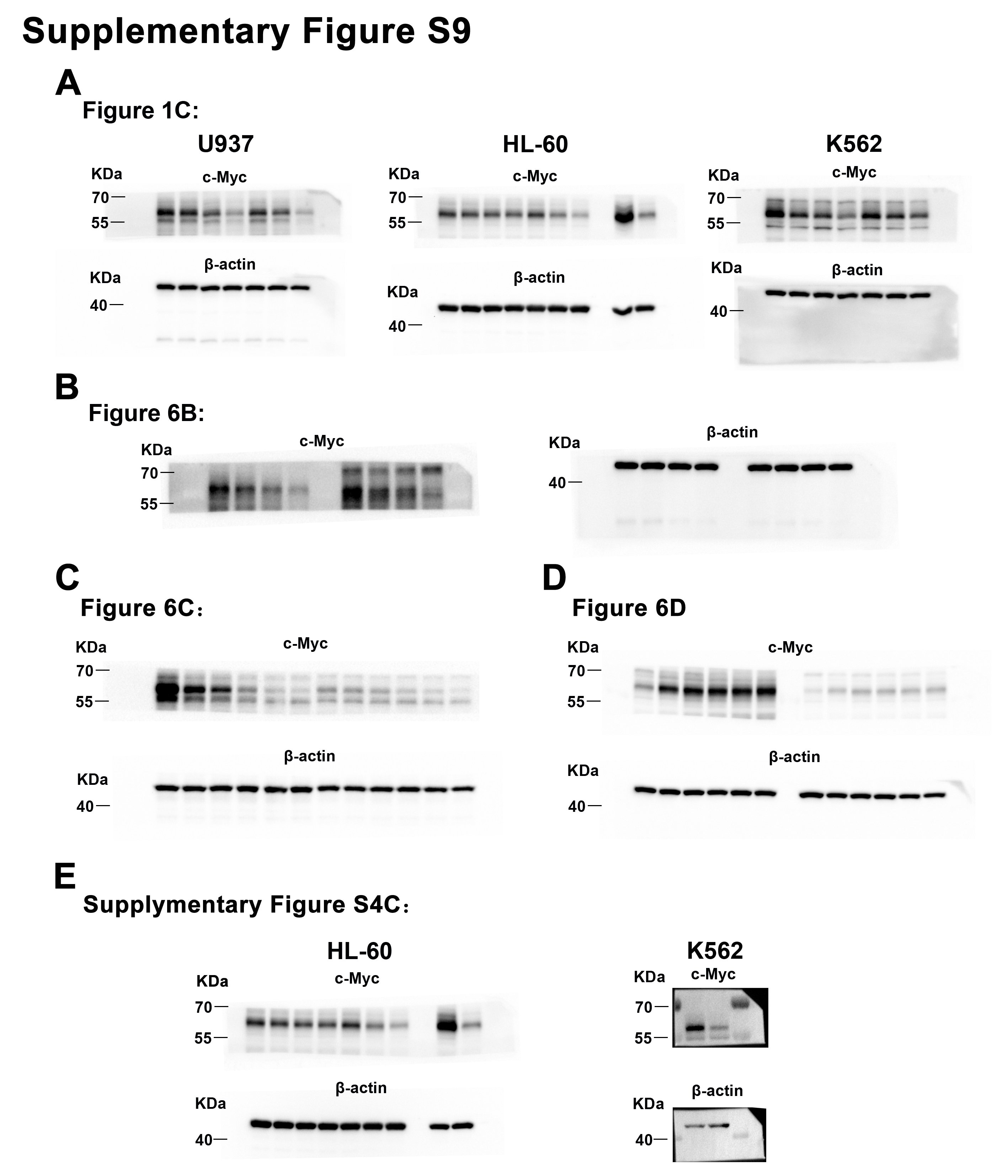

Supplement: Supplementary file 3 [file Image9.JPEG]

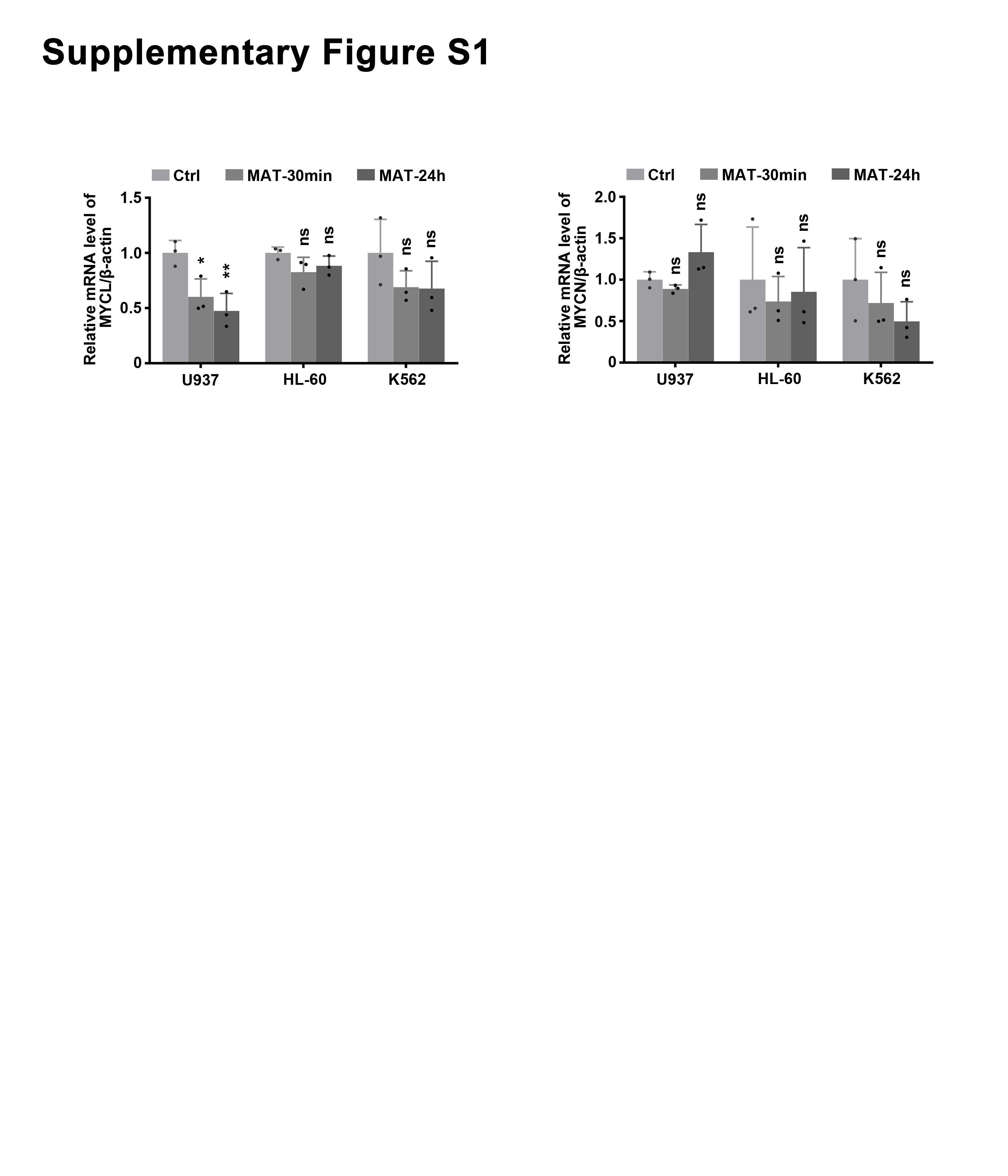

Supplement: Supplementary file 6 [file Image1.JPEG]

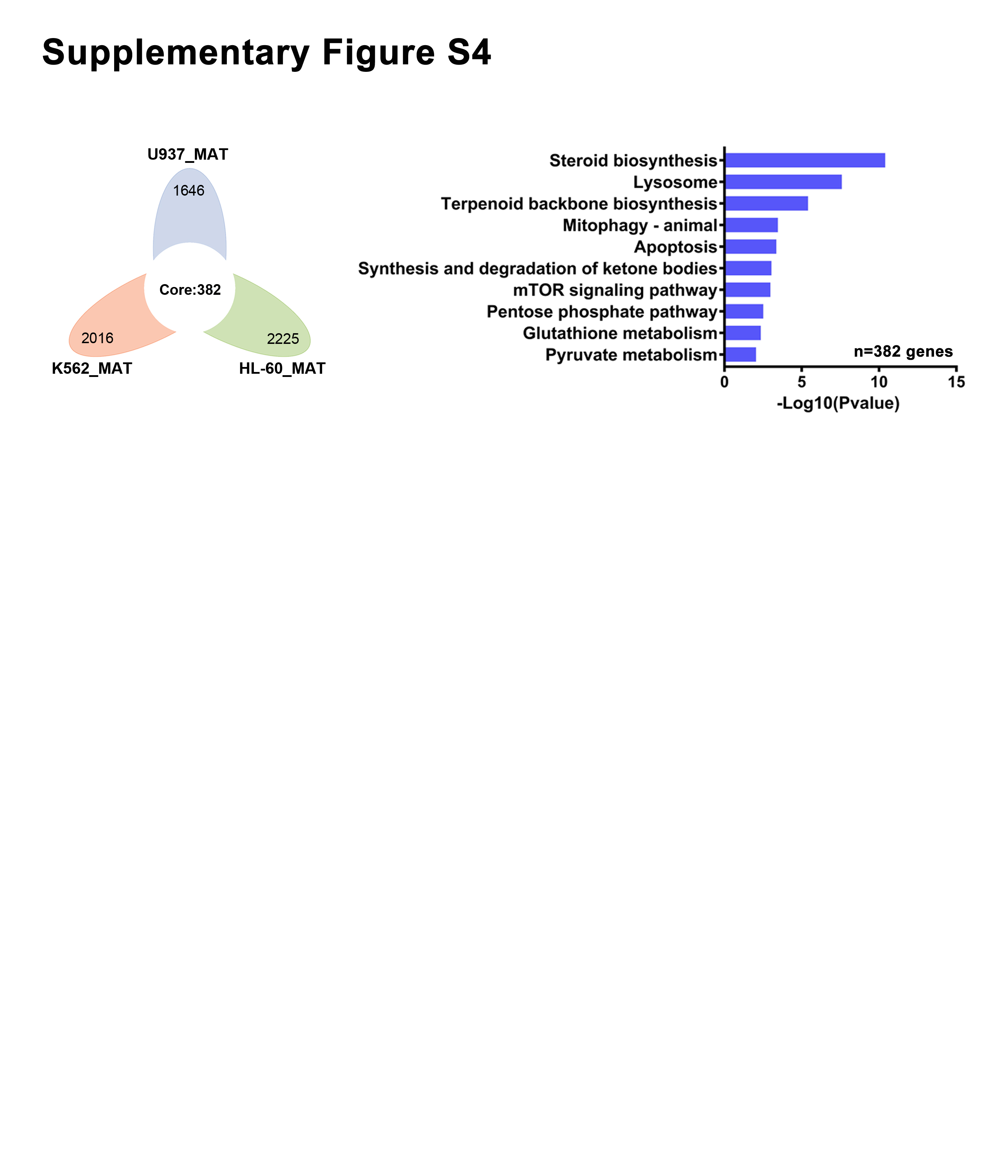

Supplement: Supplementary file 7 [file Image4.JPEG]

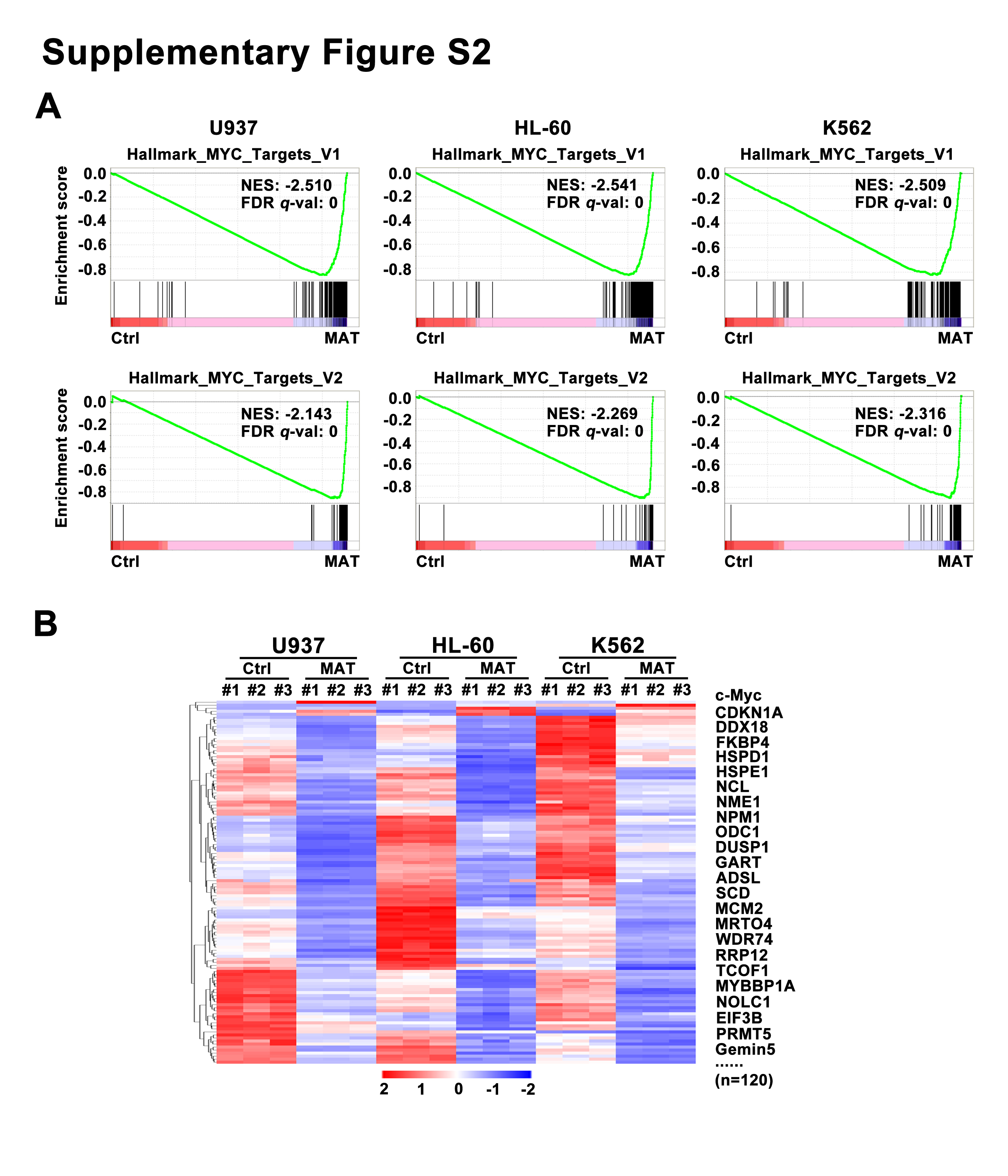

Supplement: Supplementary file 10 [file Image2.JPEG]

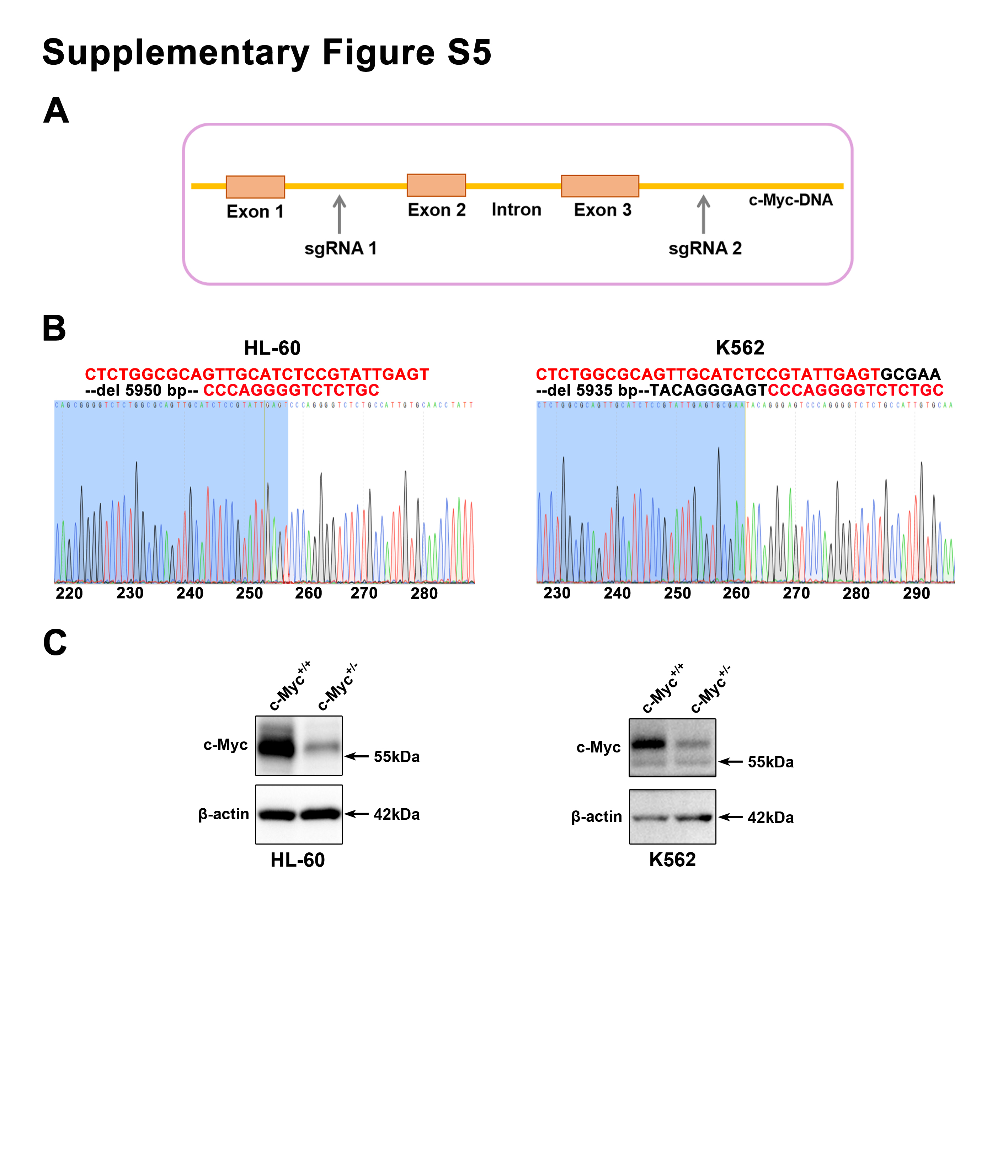

Supplement: Supplementary file 11 [file Image5.JPEG]

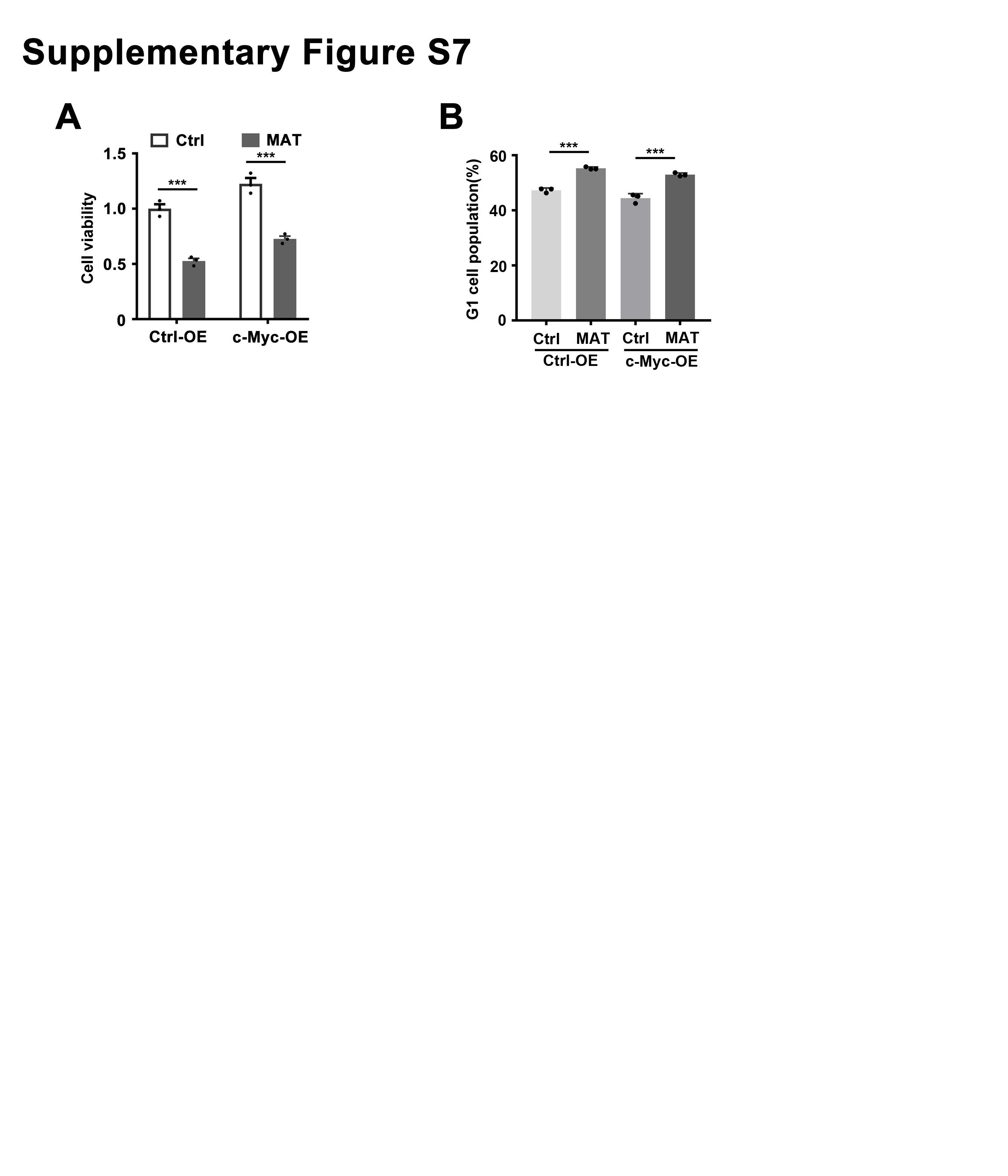

Supplement: Supplementary file 14 [file Image7.jpg]

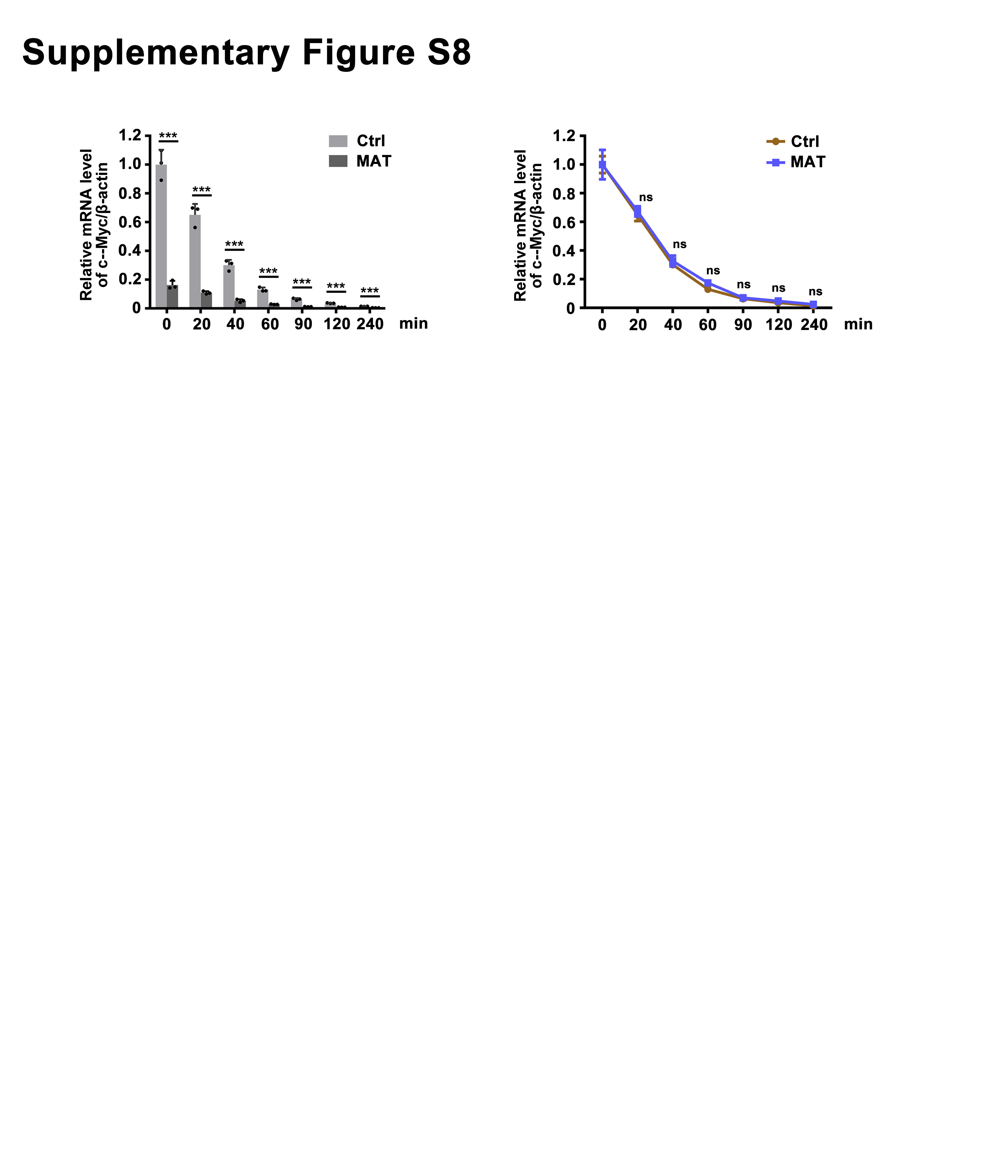

Supplement: Supplementary file 17 [file Image8.jpg]

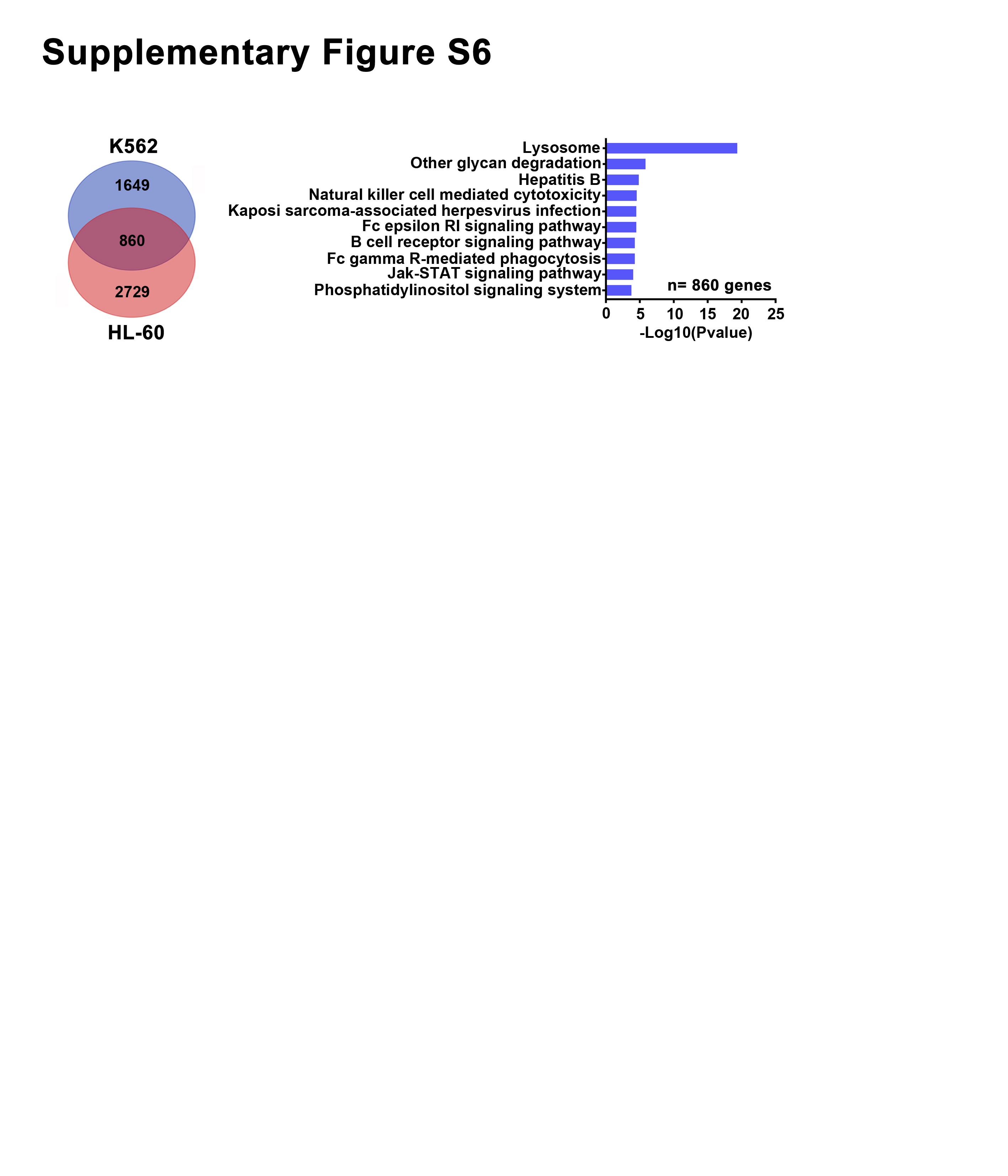

Supplement: Supplementary file 18 [file Image6.JPEG]
